# Immune Mediated Shaping of Microflora Community Composition Depends on Barrier Site

**DOI:** 10.1371/journal.pone.0084019

**Published:** 2014-01-08

**Authors:** Felix Scholz, Brian D. Badgley, Michael J. Sadowsky, Daniel H. Kaplan

**Affiliations:** 1 Department of Dermatology, Center for Immunology, University of Minnesota, Minneapolis, Minnesota, United States of America; 2 BioTechnology Institute, University of Minnesota, Minneapolis, Minnesota, United States of America; Chang Gung University, Taiwan

## Abstract

Barrier surfaces, such as the intestinal lining and the skin, are colonized by a diverse community of commensal microorganisms. Although commensal microorganisms clearly impact the host immune system, whether the immune system also shapes the commensal community is poorly understood. We used 16S rDNA deep sequencing to test whether mice with specific immune defects have an altered commensal microflora. Initially, skin swabs were obtained from wild-type and Langerhans Cell (LC) deficient mice. Despite the intimate contacts that LC make with the upper epidermis, no significant differences were observed in microbial community composition. Similarly, the skin of MyD88/TRIF^−/−^, Rag1^−/−^ and heterozygous littermate controls showed no alteration in their commensal communities. Next we examined mouth swabs and feces. We did not find a difference in the MyD88/TRIF^−/−^ mice. However, we did observe a significant shift in the microbial composition in the feces and mouths of Rag1^−/−^ mice. Thus, we conclude that the adaptive immune system modulates the microbial composition at mucosal surfaces in the steady-state but LC, adaptive immunity, and MyD88-dependent innate responses do not affect the skin microbiome revealing a major distinction between barrier sites.

## Introduction

Epithelial surfaces of the body are colonized by a complex and diverse microbiota that varies between individuals, between tissues site and even within a single individual [1]–[5]. The recent availability of germ-free mice and metagenomic approaches has begun to reveal the complex interplay between host and commensal microflora. In the intestines, gut microbiota are important for many aspects of physiology including vitamin production, nutrient absorption, and metabolic phenotype [1], [6], [7]. Dysbiosis of the microbiota has been linked to several disorders including obesity, diabetes, colorectal cancer, inflammatory bowel disease and atopic dermatitis [8]–[13].

In the intestines, it is now well documented that the presence of a microbiota as well as the presence/absence of specific commensal microorganisms influences development of the intestinal immune system. Segmented filamentous bacteria (SFB), *Candidatus svagella*, are common commensals of epithelial lining of the gut and are required for differentiation of T helper 17 (TH17) cells and also induce luminal secretion of IgA [14]–[16]. In addition, clostridial species in the distal colon and polysaccharide A derived from *Bacteroidetes* species strains induce IL-10 production and promote T-regulatory cell development [17]–[20]. Although less studied, commensal microorganisms on the skin also affect cutaneous immune responses. In humans, skin colonization with *Staphylococcus aureus* is strongly associated with flares of atopic dermatitis [8]. Studies in mice have shown that the presence of commensal bacteria suppress inflammatory responses to skin injury through a TLR2 dependent mechanism [21]. Furthermore skin microflora controls local inflammation and tunes skin-resident T cell responses through an IL-1-dependent mechanism [22]. Similarly, a product of *Staphylococcus epidermidis* enhances protection from cutaneous infection via increased expression of antimicrobial peptides [23].

The microbiota can also be affected by the host. This has been best demonstrated during intestinal inflammation. Mice with either chemically induced colitis or genetic mutations, like NLRP6 or TRUC mice that produce colitis, have a greatly altered intestinal microbiome [24]–[27]. Similarly, matriptase deficient mice, a model of ichthyosis with a defective *stratum corneum*, have an altered cutaneous microbiome [28]. Other than MyD88^−/−^ mice which have an intact intestinal microbiome, the ability of host immunity to shape the microbiome at barrier surfaces in the absence of barrier disruption has not been explored [29].

Numerous immune cells reside in the skin. Langerhans cells are antigen-presenting cells that reside in the epidermis where they are able to acquire antigen from the epidermis and *stratum corneum* [30]. Memory CD4 and CD8 αβ T cells can be found in the in the dermis along with γδ T cells which are also found in high numbers in the epidermis [31], [32]. These cell types have the capacity to elicit antimicrobial responses through the secretion antimicrobial peptides (e.g. cathelicidin) and elaboration of cytokines that enhance keratinocyte proliferation (e.g. IL-22) and recruitment of neutrophils (e.g. IL-17) [33]–[36].

To determine whether components of the immune system actively control the composition of commensal communities at barrier surfaces, we used 16S rDNA deep sequencing to determine the microbial community composition of the skin, mouth, and intestines in mice with isolated defects in intraepithelial dendritic cells (Langerhans cells), adaptive immunity (Rag1^−/−^) and innate immunity (MyD88/TRIF^−/−^).

## Results

### Langerhans cells do not shape the skin microbiome

To test whether the absence of Langerhans Cells (LC) in the epidermis affects the composition of bacterial commensal communities on the skin, we obtained skin swabs from the ears of huLangerin-DTA mice [37]. These mice have a constitutive and complete absence of LC from birth. Since vertical transmission strongly affects the composition of the microbiome [29], we also swabbed control, transgene negative, wild-type littermates (LMC) that were housed in the same cage as transgene positive huLangerin-DTA mice under specific pathogen free conditions.

**Figure 1:**
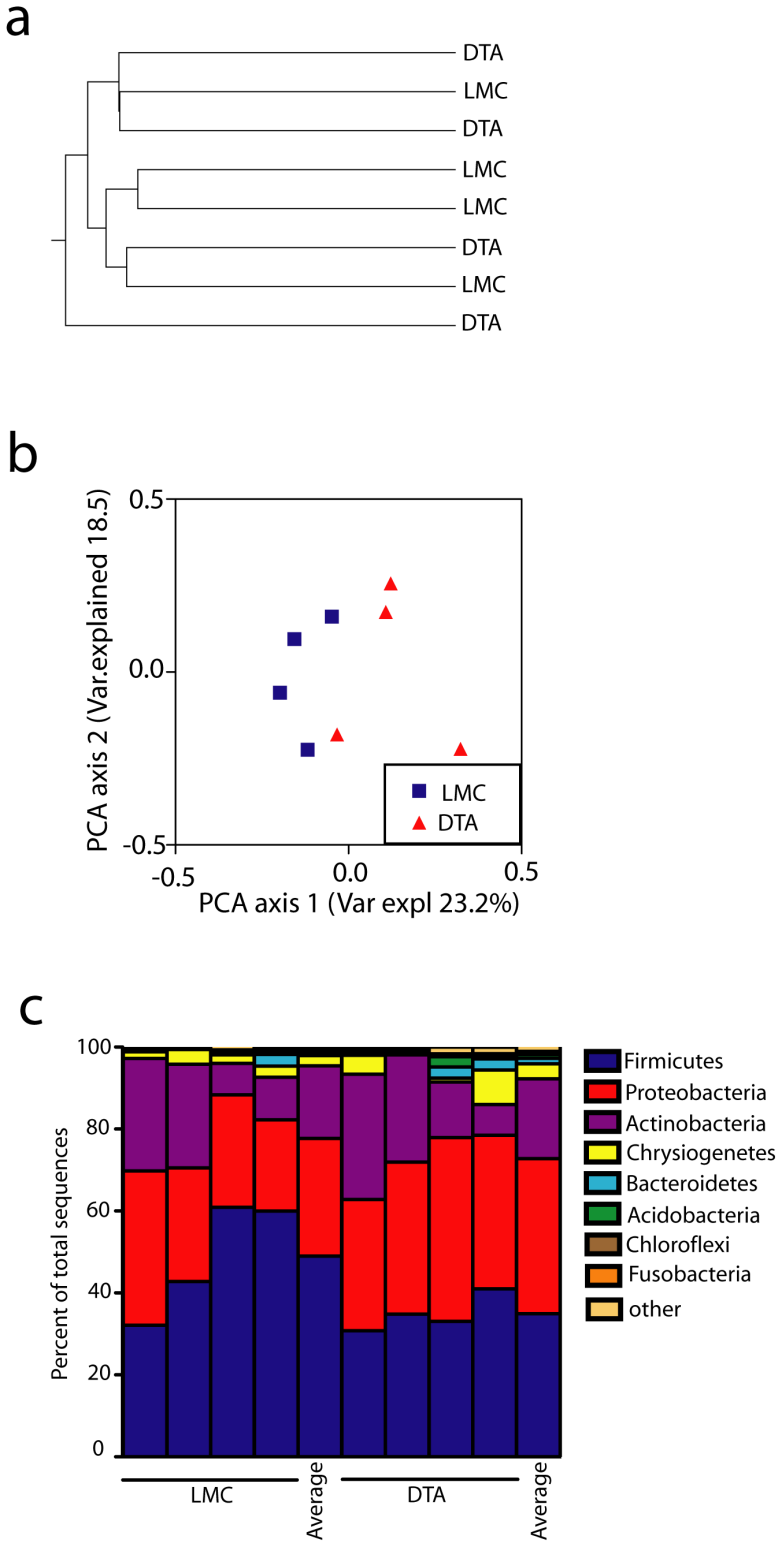
Langerhans cell deficient mice have an unaltered skin microbiome. a) Distance tree based upon Unweighted Paired Group Method with Arithmetic Mean (UPGMA) clustering of unweighted UniFrac distances among microbiomes of littermate controls (LMC) and huLangerin-DTA mice (DTA) (n = 4). No significant differences based on Analysis of Similarity (ANOSIM) were observed. b) PCoA analysis of OTU from skin of DTA and littermate LMC. c) Bacterial Phyla distribution on ear skin of DTA mice and LMC is shown. Each symbol represents data from an individual animal.

DNA isolated from the swabs was PCR amplified using primers that span the V6 hypervariable region of the bacterial 16S ribosomal DNA (rDNA) gene. The amplicons were sequenced, screened and quality sorted and clustered into operational taxonomic units (OTUs) at a cutoff of 90%. The number of OTUs per sample ranged between 414 and 572. Taxonomy was assigned to OTU consensus sequences and the unweighted UniFrac distances were calculated between all sample pairs [38]. To determine whether different community compositions were present in huLangerin-DTA and littermate control mice, we compared the groups using Analysis of Similarity (ANOSIM) and found no significant difference (p = 0.263). To visualize microbiome differences among samples, we constructed a community structure distance tree and principal coordinate analyses (PCoA) based on unweighted Unifrac (Figure 1a and 1b). Samples from control and huLangerin-DTA mice did not appear to cluster.

The Shannon diversity index, a measure of microbial diversity, was similar between the two groups (Figure S1a). As expected, *Firmicutes*, *Proteobacteria*, *Actinobacteria*, and low amounts of *Bacteroidetes* were observed on the skin which is consistent with prior reports [28]. The distribution of individual phyla (Figure 1c) and families (data not shown) between control and huLangerin-DTA mice appeared similar. Thus, the skin microbiome does not differ significantly between WT and huLangerin-DTA mice indicating that the absence of Langerhans cells does not affect the overall community composition among skin commensal bacteria.

### Adaptive Immunity does not shape the skin microbiome

To determine whether skin resident T cells (e.g. dendritic epidermal T cells and both TCRαβ and γδ T cells in the dermis) or humoral responses have the capacity to alter the commensal communities of the skin, we compared the microbiome from ear swabs of Rag1^−/−^ and control Rag1^−/+^ mice. Wild-type C57BL/6 mice (littermate controls from huLangerin-DTA matings) were bred with Rag1^−/−^ mice. The resulting heterozygous F1 mice were backcrossed with Rag1^−/−^ mice. Cohorts were generated using Rag1^+/−^ and Rag1^−/−^ littermates. As above, ear skin of these cohorts were swabbed for sequencing. As an additional control, ear skin swabs from the parental homozygous Rag1^−/−^ used to establish the cohorts were also obtained.

**Figure 2:**
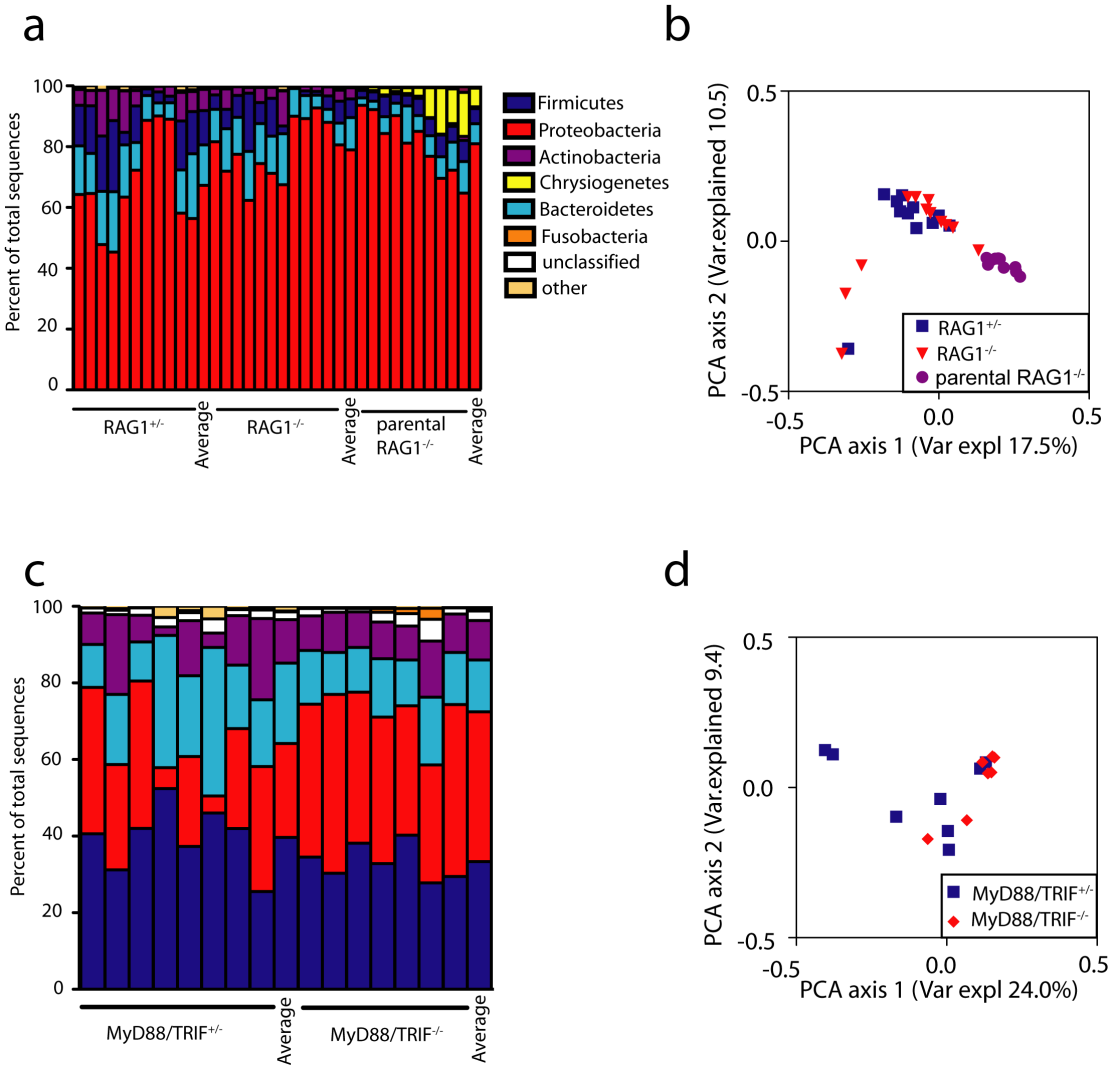
Rag1^−/−^ and MyD88/TRIF^−/−^ mice have an unaltered skin microbiome. a) Phyla distribution and (b) PCoA analysis (based on unweighted UniFrac distances among samples) of microbiomes from the skin of Rag1^−/−^(n = 12), Rag1^+/−^(n = 11) and parental Rag1^−/−^ (n = 10) mice. OTU from Rag1^−/−^ and Rag1^+/−^ littermates differed from parental Rag1^−/−^ mice (ANOSIM, p<0.001) but not from each other. c) Phyla distribution and (d) PCoA analysis of bacterial sequences obtained from MyD88/TRIF^+/−^ (n = 8) and MyD88/TRIF^−/−^ (n = 7) ear skin. No significant differences based on ANOSIM were observed.

Using the same approach described above, we found that microbiomes from Rag1^−/−^ and Rag1^+/−^ mice displayed similar phyla distribution and clustered together by PCoA with no significant differences (ANOSIM, p = 0.348) (Figure 2a and 2b). Shannon diversity scores were also similar between the groups (Figure S1a). Notably, samples from parental Rag1^−/−^ mice did cluster and differed significantly from intercrossed Rag1^−/−^ and Rag1^+/−^ mice (ANOSIM, p<0.001) highlighting the importance of vertical transmission in determining commensal community structure. Thus, despite the ability to detect differences in commensal community between independently housed animals, we did not observe any alteration in the microbial community composition in Rag1^−/−^ vs. Rag1^+/−^ mice indicating that cells of the adaptive immune system do not affect the skin microbiome. Interestingly, the observed phyla in Rag1^−/−^ and Rag1^+/−^ mice were dominated by *Proteobacteria* and *Bacteroidetes* with fewer *Firmicutes* and *Actinobacteria*. This is distinct from the distribution of phyla seen in huLangerin-DTA cohorts and closely resembled the phyla seen in the parental Rag1^−/−^ mice used to establish the cohort (Figure 2a). Thus, the composition of the skin microbiome is not fixed and can vary considerably between independent lines of mice highlighting the importance of using littermate mice as controls.

### MyD88/TRIF dependent innate responses do not shape the skin microbiome

To determine whether engagement of Toll-like receptors (TLR) and the inflammatory mediators IL-1, IL-18, IL-33 and IL-36α affect skin commensal microbial communities, we compared the skin microbiome of MyD88/TRIF^−/−^ mice with that of MyD88/TRIF^+/−^ controls. We employed the same breeding strategy used for Rag1^−/−^ mice to generate cohorts of MyD88/TRIF^−/−^ mice and MyD88/TRIF^+/−^ littermate controls. Microbiomes from MyD88/TRIF^−/−^ and MyD88/TRIF^+/−^ mice clustered together by PCoA and were not significantly different (ANOSIM, p = 0.348) (Figure 2d). The diversity index was also similar between the groups (Figure S1a). The proportion of sequences belonging to the phyla *Bacteroidetes* were somewhat increased compared with the Rag1^−/−^ and huLangerin-DTA cohorts in this independently maintained mouse line with but was similar between MyD88/TRIF^−/−^ and MyD88/TRIF^+/−^ controls (Figure 2c). Thus, the global absence of sensitivity to TLR-ligands, MyD88-dependent cytokines does not alter the community composition of the skin microbiome.

### Adaptive but not innate immunity shapes the oral and colonic microbiome

**Figure 3:**
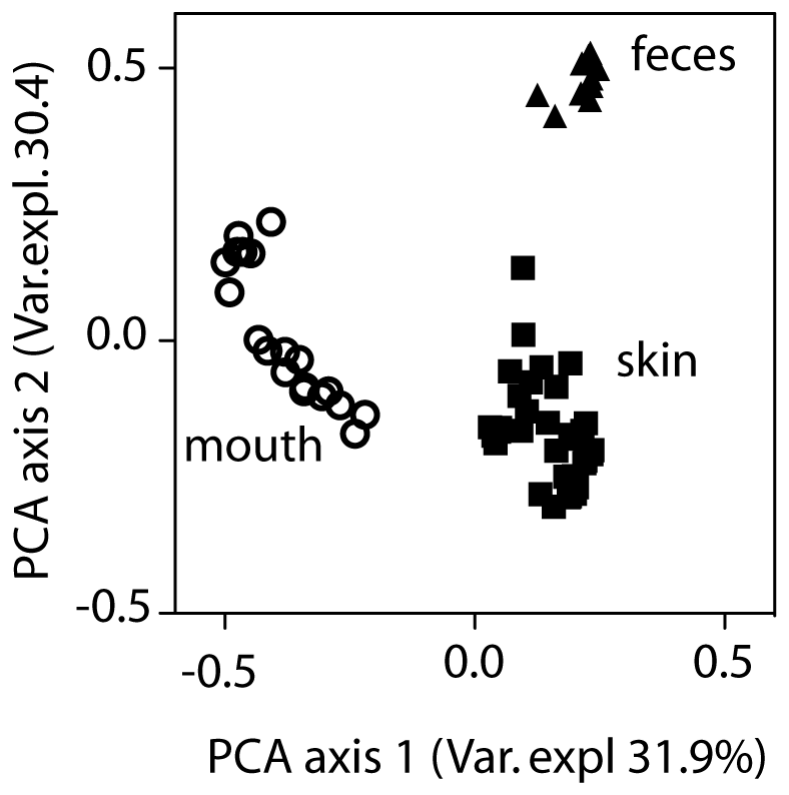
The microbiomes of skin, mouth and colon are distict. Principle coordinates analysis of unweighted UniFrac distances among microbiomes from feces, skin and mouth isolated from all mice examined is shown. All 3 groups differed significantly from each other by ANOSIM (p<0.001).

**Figure 4:**
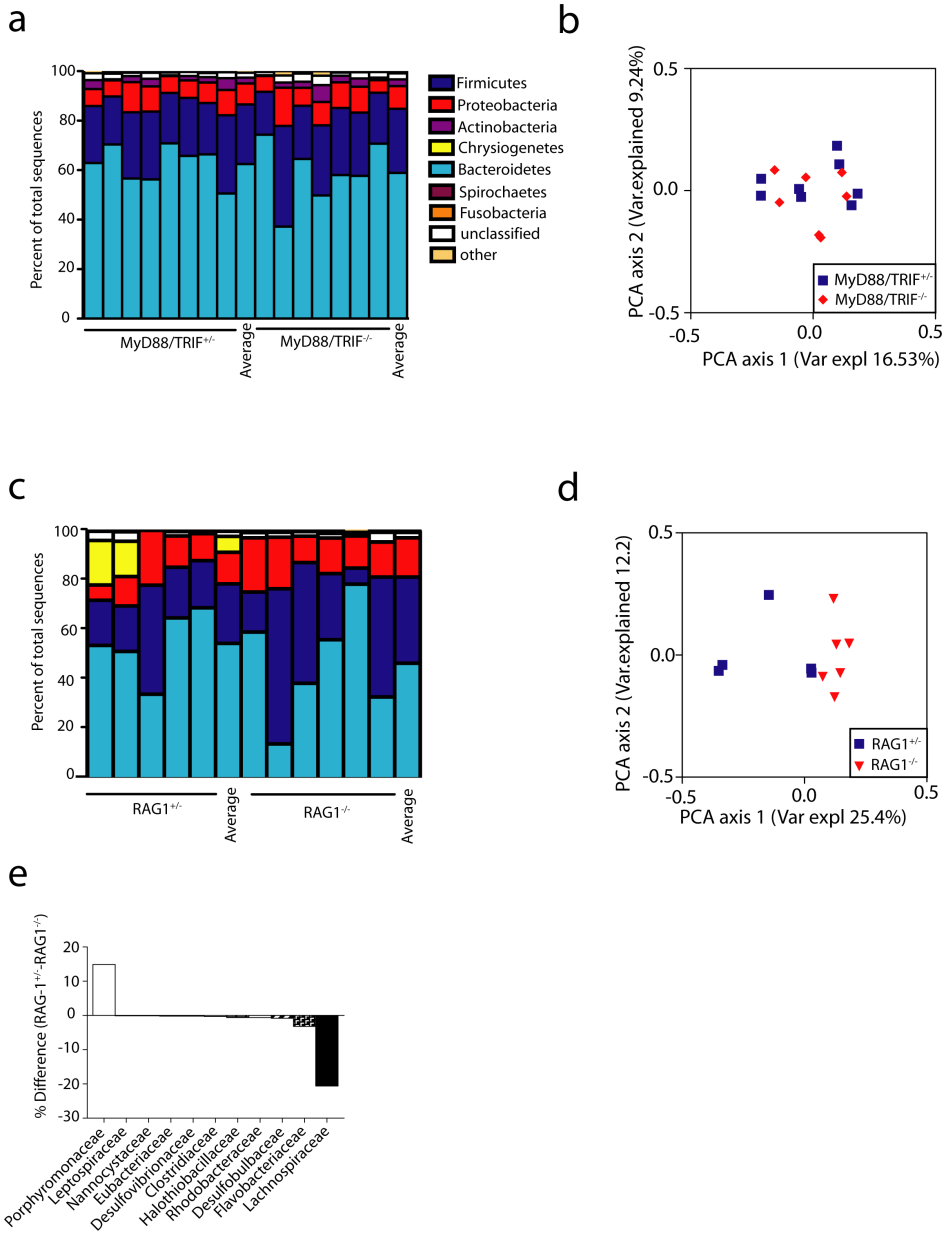
The colonic microbiome is altered in Rag1^−/−^ but not MyD88/TRIF^−/−^ mice. (a) Phyla distribution and (b) PCoA analysis (based on unweighted UniFrac distances among samples) of microbiomes from fecal pellets of MyD88/TRIF^+/−^ (n = 8) and MyD88/TRIF^−/−^ (n = 7) mice is shown. No significant differences based on ANOSIM were observed (c) Phyla distribution and (d) PCoA analysis from Rag1^−/−^ (n = 6) and Rag1^+/−^ (n = 5) fecal pellets is shown. Community composition differs between Rag1^+/−^ and Rag1^−/−^ based on ANOSIM, p = 0.004. (e) Comparison of bacterial family abundances between RAG1^−/−^ and RAG1^+/−^, differences are shown in percent.

To determine whether the composition of the microbiome is also independent of immune status at other barrier sites, we obtained oral swabs and fecal samples from the same cohorts of Rag1^−/−^, MyD88/TRIF^−/−^, and control mice used above. As expected, PCoA analysis of OTUs pooled from all mouse genotypes revealed strong clustering based on site and highly significant differences in microbiome composition were detected (ANOSIM, p<0.001) (Figure 3). *Bacteroidetes* and *Firmicutes* were the dominant phyla in feces (Figure 4a). As was seen in the skin, diversity (Figure S1b), the distribution of individual phyla (Figure 4a) and community composition (Figure 4b) were similar in the feces of MyD88/TRIF^−/−^ and MyD88/TRIF^+/−^ mice.

In contrast, PCoA analysis of OTUs revealed modest clustering of samples obtained from Rag1^−/−^ vs. Rag1^+/−^ mice and a significantly altered community composition (ANOSIM, p = 0.004) (Figure 4d). This was largely driven by an over representation of members of the family *Lachinospiraceae* in the phylum *Firmicutes* in Rag1^−/−^ mice (Figure 4e and Figure S2). Concomitantly, members of the family *Porphyromonadaceae* were relatively less abundant in the Rag1^−/−^ than the Rag1^+/−^ mice. Modest differences between Rag1^−/−^ and Rag1^+/−^ at the phyla level were also observed (Figure 4c).

**Figure 5:**
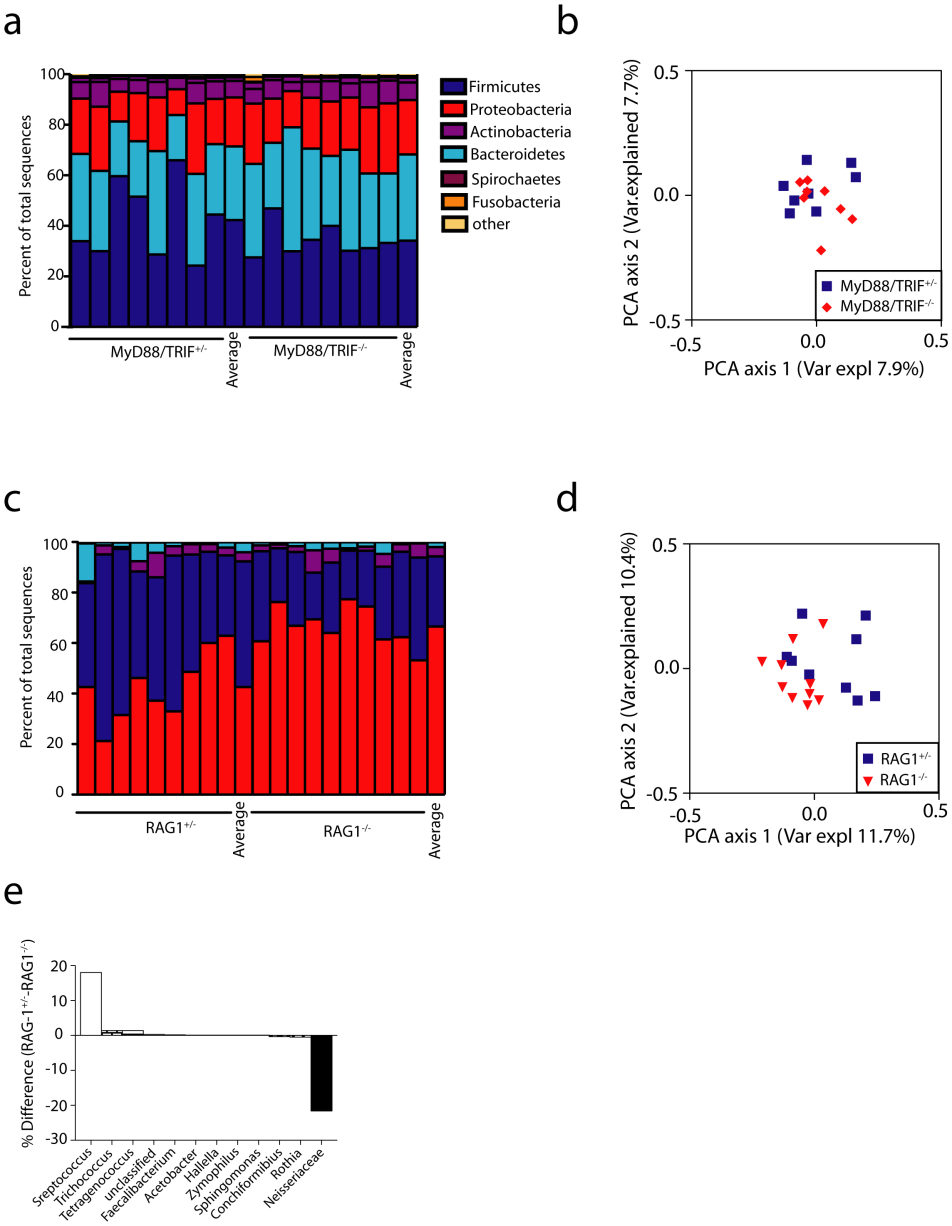
The oral microbiome is altered in Rag1^−/−^ but not MyD88/TRIF^−/−^ mice. (a) Phyla distribution and (b) PCoA analysis (based on unweighted UniFrac distances among samples) of microbiomes in oral swabs of MyD88/TRIF^+/−^ (n = 8) and MyD88/TRIF^−/−^ (n = 8) mice is shown. No significant differences based on ANOSIM were observed. (c) Phyla distribution and (d) PCoA analysis is shown from Rag1^−/−^ (n = 10) and Rag1^+/−^ (n = 9) oral swabs. Community composition differs between Rag1^+/−^ and Rag1^−/−^ based on ANOSIM, p = 0.013. (e) Comparison of bacterial family abundances between RAG1^−/−^ and RAG1^+/−^, differences are shown in percent.

Similar results were obtained by examination of the oral microbiome. The distribution of phyla was similar between MyD88/TRIF^−/−^ and MyD88/TRIF^+/−^ mice, ANOSIM analysis was not significant (p = 0.63), and in PCoA visualization OTUs samples from both genotypes clustered together (Figure 5a and b). In contrast, the community composition of the oral microbiome was significantly different in Rag1^−/−^ compared to Rag1^+/−^ mice (ANOSIM, p = 0.013) (Figure 5C, D). The taxanomic shift, however, was different in the oral samples, with the families *Neisseriaceae* being significantly more abundant and the *Streptococcaceae* being less abundant in Rag1^−/−^ mice (Figure 5e and Figure S2b). Thus, the adaptive, but not innate, immune system contributed to a modest alteration of the composition of the commensal community in both the colon and oral cavity.

## Discussion

Here we report that the overall composition of the microbiome on the skin of mice is not significantly affected by the loss of Langerhans cells, MyD88/TRIF signaling or the adaptive immune system. The diversity of commensal bacteria on the skin was similar in mice with a constitutive absence of epidermal Langerhans cells (huLangerin-DTA), mice that lack all B and T cells (Rag1^−/−^), and mice in which all cells are unable to respond to TLR-agonists as well as the pro-inflammatory cytokines IL-1, IL-18, IL-33, and IL-36α (MyD88/TRIF^−/−^). These data demonstrate that recognition of bacterial products via TLRs is not an obligate component determining the composition of the skin microbiome under steady-state conditions. The generation of active IL-1β and IL-18 resulting from inflammasome activation is also redundant. These data also demonstrate that local immune cells such as epidermal LC and dendritic epidermal T cells (DETC) as well as dermal T cell populations that could potentially sense the presence of commensal bacteria through other pathways are also redundant. The presence of skin commensal microorganisms regulates and enhances cutaneous immune responses [22], [39]. The converse, however, does not appear to occur. The composition of the skin microbiome is presumably shaped by factors controlling the local environmental niche but not by innate or adaptive immune effectors [3].

The observed similarity of skin microflora in mice with immune defects does not result from our inability to detect differences in microbial flora. We observed distinct microbial community compositions in independently maintained strains that were determined by husbandry. For example, the parental Rag1^−/−^ mice used to generate cohorts of Rag1^+/−^ and Rag1^−/−^ mice had skin flora dominated by *Proteobacteria* which was less prominent in huLangerin-DTA and MyD88/TRIF mice. This highlights the importance of having used littermate controls for these studies. Importantly, the microbiome of mice derived from the same matings were relatively stable. Thus, we are able to conclude that inter-individual variation is greater than the potential effects of any of the immune defects examined. We can not exclude that Langerhans cells, T and B cells or MyD88/TRIF could effect the microbial composition of the skin outside of special pathogen free husbandry conditions.

The microflora found in the colon, mouth, and on the skin was highly divergent as has been previously reported [16]. Of note, the colonic bacterial flora was only minimally affected by husbandry conditions and was quite stable between individuals and between independent matings. In contrast, as was seen with skin, the oral flora was more variable between mouse lines. Interestingly, even though mice are well known to be coprophagic, the oral flora was quite distinct from the colonic flora.

Unlike skin, the microbiome in both the mouth and colon was altered in Rag1^−/−^ mice compared with littermate controls. This has recently also been reported with singly housed Rag1^−/−^ and CD45^−/−^ mice [40]. Both the oral and colonic mucosae have dense networks of submucosal effector TCRαβ and TCRγδ that are absence in Rag1^−/−^ mice. IgA is also actively secreted into both sites [41], [42]. One of the clearest alterations in the flora of Rag1^−/−^ mice was an increase in the number of fecal *Lachnospiraceae*. This family of bacteria which is in the class *Clostridia* and phylum *Firmicutes*, contains a number of immunologically important species, including segmented filamentous bacteria which are required for Th17 differentiation [43]. Notably, the number of Clostridia was found to be increased in the colon of AID^−/−^ mice that cannot generate IgA [44]. Although the fecal microbiome in AID^−/−^ mice was not analyzed by deep sequencing and the oral microbiome was not examined, the similarities with our data suggests that the absence of IgA in Rag1^−/−^ mice may contribute to the observed alterations.

In summary, the interplay between the immune system and commensal microorganisms that occurs in skin is quite different from the interplay in the mouth and colon. In the skin, commensalism is not affected by immune effectors while commensalism at oral and intestinal mucosae is shaped by adaptive immunity. This demonstrates a fundamental immunological difference between these barrier sites. The skin may be unique due to the presence of the *stratum corneum* since commensal communities on skin are altered when the barrier is disrupted [8], [28], [45]. Our studies have focused on steady-state conditions. In the future it will be important to ascertain whether immune effectors have the capacity to modulate colonization of inflamed and/or wounded skin.

## Materials and Methods

### Mice

All mouse procedures were performed with approval of the University of Minnesota Institutional care and use committee (IACUC # 1012A93332). All mice used for experiments were housed under specific pathogen-free conditions. The huLangerin-DTA mice were bred heterozygous and animals from the same litters and cages were used for experiments. To generate comparable Rag1^−/−^ mice we crossbred wild-type C57BL/6 mice originating from the huLangerin-DTA line to Rag1^−/−^ to avoid parental biases. The resulting heterozygous F1 Rag1^+/−^ mice were used for further breeding to gain Rag1^+/−^ and Rag1^−/−^ from same litters for experiments. MyD88^−/−^/TRIF^−/−^ mice were bred with wild-type C57BL/6 females from the huLangerin-DTA line. The F1 generation was crossed to gain MyD88^+/−^/TRIF^+/−^ and MyD88^−/−^/TRIF^−/−^ mice from same parental cages for experiments. 8 to 10 weeks old mice were used for cohorts.

### DNA extraction

Three types of samples were obtained over the course of the study. Skin and mouth samples were taken by vigorously swabbing the ear or oral cavity of the mouse, respectively. Swabs (Epicentre, Madison,WI) were soaked in a buffer solution of sterile PBS 1% TritonX before use. After sampling, the swabs were digested with 25 U/µl achromopeptidase (WAKO, Richmond,VA) for 30 minutes to lyse cells. Buffer soaked but unused swabs were also digested as controls. Stool samples were obtained via the collection of fecal pellets from the colon, from which DNA was extracted using a MOBIO PowerSoil DNA extraction kit (MOBIO, Carlsbad, CA), according to the manufacturer's instructions. Approximately 0.25 to 0.50 grams of fecal material was used as input material for extraction. All DNA samples were stored frozen at −20°C until amplified.

### Amplification

The V6 hypervariable region of the bacterial 16S rDNA was amplified for sequencing in this study. In the initial portion of the study, focusing on huLangerin-DTA mice, a single set of V6 primers was used [46]. For the remainder of the study, this primer set was expanded to include additional degeneracies that increase the universality of the primers [47]. All primer sequences included a 6 bp ID tag on the 5′ end of either the forward or reverse primer(s) that is specific to each sample to allow for multiplexed sequencing [48]. Amplifications were performed in triplicate 50 µl reactions. Each reaction included 10 µl of Takara PrimeStar PCR Buffer, 2 µl each forward and reverse primer (10 µM concentration), 10 µl of template, 0.5 µl of Takara PrimeStar polymerase and 21.5 µl of water. Thermal cycling conditions included 3 min at 95, 32 cycles of 30 sec at 95, 30 sec at 55, and 30 sec at 72 for 32 cycles, followed by a final extension step of 3 min at 72. Amplification products were visualized using gel electrophoresis to confirm amplification of properly sized products. Control samples were included and did not produce a PCR product. Triplicate reactions were purified individually using the Qiaquick PCR Purification Kit (Qiagen, Valencia, CA) or Qiaquick Gel Extraction Kit (Qiagen, Valencia, CA). Purified DNA concentrations were measured using the QuBit High Sensitivity DNA quantification system (Invitrogen, Carlsbad, CA) and stored at −20°C until sequencing.

### Sequencing

Three sequencing runs were performed for this study. For each sequencing run, equimolar aliquots of the purified amplicon library from each sample were pooled to give ∼1 µg of DNA in a 100 µl total volume. Final pooled DNA concentration was measured by Quant-IT PicoGreen quantitation (Invitrogen, Carlsbad, CA). Amplicon size analysis was done using an Agilent DNA 1000 chip and a 2100 BioAnalyzer (Agilent, Santa Clara, CA) to ensure no undesired products were in the pool. The pooled samples were then submitted frozen to the sequencing center for library preparation and sequencing on the Illumina HiSeq 2000 platform with 25% Phi-X added to increase sequence diversity (Caporaso et al. 2012). The first sequencing run (8 pooled samples) was conducted by the National Center for Genome Resources (Santa Fe, NM, USA) and the second and third sequencing runs were conducted by the University of Minnesota Biomedical Genomics Center (St. Paul, MN, USA). The first sequencing run yielded in a total of 6.8 million reads with a post filter average of 357,666 reads per sample. The second run yielded in 47 million reads with a post filter average of 408,316 reads per sample and the third run yielded 108 million reads and a post filter average of 1.2 million reads per sample. Each sequencing run represented an independent set of experimental samples. Data from different runs were not compared to avoid confounding results with any potential variation between primer sets or sequencing facilities.

### Sequence Processing and Analysis

Sequence data were processed and analyzed using MOTHUR [49]. Data were first filtered for quality to exclude sequences containing any ambiguous bases, homopolymers >7 bp, mismatches in the primers or ID codes, or a quality score that averaged below 35 in any 50 bp window [50]. To reduce sequencing and amplification noise, sequences that appeared only one time in the entire sequencing run were also removed from the analysis, and then sequences that differed by only 1 bp were preclustered [50], [51]. The remaining high quality sequences were aligned to the RDP7 16s database [52] and any sequences which did not align well to the V6 region were removed. Next, chimeric sequences were detected using UCHIME [53] and removed from subsequent analysis. Finally, remaining sequences were classified and any sequences that represented mitochondrial or chloroplast DNA were removed.

Prior to analysis, a random subset of sequences were chosen from each sample to match the lowest number among all samples in order to balance sampling effort and ensure comparable diversity measures. In the first and second sequencing runs, 41,500 sequences were selected, and 100,000 were selected in the third. Sequences were then clustered into OTUs at a cutoff of 90% similarity to avoid overestimating diversity [54], [55], although the sequences were also analyzed based upon OTUs clustered at 97% similarity with similar results and conclusions. Taxonomy was assigned to OTU consensus sequences using the RDP7 database using the Bayesian method with a bootstrap algorithm (100 iterations) and a probability cutoff of 0.60. From OTU abundance data, rarefaction curves, Shannon diversity indices, and Chao1 richness estimates were calculated.

For beta diversity, similarities in community compostions among samples were calculated using the unweighted UniFrac distance metric on the Fast UniFrac website [38], [56]–[58]. UniFrac distances were used to construct principal coordinate analyses for data visualization and also to test for significant differences community structure using ANOSIM, which was run through the mothur software package. Significant differences in taxonomic groups were analyzed using the Metastats software, which includes correction for multiple comparisons [59].

Sequence data are available in the NCBI Short Read Archive as BioProject PRJNA226001.

## Supporting Information

Figure S1 **Commensal diversity varies by husbandry but not genotype.** Shannon diversity indices of microbiomes from litter mate controls (LMC), huLangerin-DTA (DTA), Rag1^+/−^,Rag1^−/−^, MyD88/TRIF^+/−^ and MyD88/TRIF^−/−^ obtained from (a) ear skin swabs (b) fecal pellets and (c) oral swabs. No statistical differences between littermates within cohorts of individual experiments have been detected.
(TIF)

Figure S2 **Family composition of feces and oral microbiome is altered in Rag1^−/−^ mice.** Bacterial families showing the greatest difference between Rag1^+/−^ (n = 9) and Rag1^−/−^ (n = 8) mice is shown based on analysis of OTUs observed in the feces (a) or mouth (b). The percentage of total sequences from each sample belonging to the indicated family, significance and percent difference are shown.
(TIFF)
