# Supplementary figures and images for: Immune Mediated Shaping of Microflora Community Composition Depends on Barrier Site
Source: PLoS One. 2014 Jan 8;9(1):e84019. doi: 10.1371/journal.pone.0084019 (PMC3885526; doi:10.1371/journal.pone.0084019)

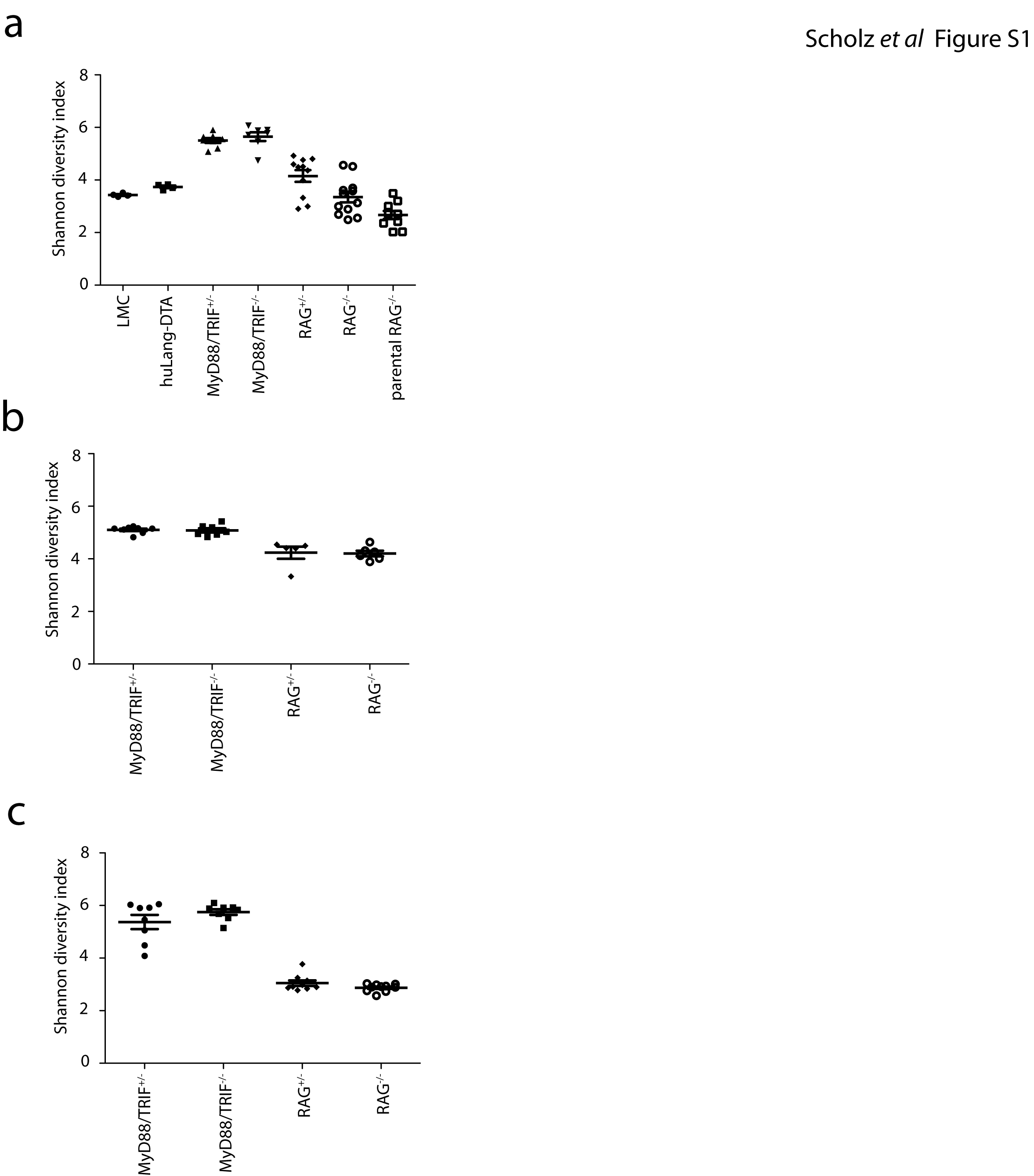

Supplement: Figure S1 — Commensal diversity varies by husbandry but not genotype. Shannon diversity indices of microbiomes from litter mate controls (LMC), huLangerin-DTA (DTA), Rag1+/−,Rag1−/−, MyD88/TRIF+/− and MyD88/TRIF−/− obtained from (a) ear skin swabs (b) fecal pellets and (c) oral swabs. No statistical differences between littermates within cohorts of individual experiments have been detected. (TIF) [file pone.0084019.s001.tif]
